# Vascular Tissues Distribution Affects Calcium and Calcium Oxalate Crystals in Fruits of Wild Tomato (*Lycopersicon pimpinellifolium* (L.) Mill.)

**DOI:** 10.3390/plants12223893

**Published:** 2023-11-18

**Authors:** Élder Antônio Sousa Paiva, Cleber Cunha Figueredo, Hermínia Emília Prieto Martinez

**Affiliations:** 1 Departamento de Botânica, Instituto de Ciências Biológicas, Universidade Federal de Minas Gerais, Belo Horizonte 31270-901, Minas Gerais, Brazil; cleberfigueredo@ufmg.br; 2 Departamento de Agronomia, Universidade Federal de Viçosa, Viçosa 36570-000, Minas Gerais, Brazil; herminia@ufv.br

**Keywords:** blossom-end rot, calcium distribution, calcium oxalate, calcium regulation, calcium-related disorders, tomato disorders, xylem, wild tomato

## Abstract

Tomato fruit is an excellent model for evaluating calcium regulation in plants since it expresses symptoms of either calcium deficiency or calcium excess. Aiming to evaluate the structure of the vascular system and its interactions with calcium and calcium oxalate crystals (CaOx), fruits of *Lycopersicon pimpinellifolium* were studied. Calcium levels were evaluated in basal, median, and distal pericarp portions, which were also analyzed under a light microscope to describe the structure. The *L. pimpinellifolium* pericarp shows idioblasts with calcium oxalate crystals. Vascular bundles of the basal pericarp show large transverse sections and abundant xylem vessels. The vascular bundles were smaller in the distal pericarp, and the xylem showed fewer and narrower vessels. The terminal bundles often consisted exclusively of phloem. Despite the differences observed in vascular bundle composition, the density of the vascular system was uniform in the pericarp as a consequence of bundle ramifications that occur at distal portions. The calcium concentration and crystal idioblasts decrease towards the apex of the fruit. The reduction in the xylem:phloem ratio seems to determine the low calcium concentration in the distal fruit portion.

## 1. Introduction

Calcium deficiency is rare in nature, but several Ca-deficiency disorders occur in horticulture [1], such as blossom-end rot (BER) in fruits. These disorders arise when Ca^2+^ availability is momentarily insufficient for developing tissues, occurring, among others, in tissues preferentially fed by the phloem, such as BER in tomato fruits [2].

Although calcium-deficiency disorders are well studied, there are reports of damage caused by a calcium excess, such as those promoted by the formation of calcium oxalate crystals (CaOx) in tomato fruits. In plants that precipitate calcium oxalate, there is a proportional increase in Ca^2+^, and the oxalate concentration accompanying the external calcium increases [3,4]. In tomato fruit, a symptom of calcium excess is the presence of tiny yellowish flecks, the “gold specks”, in the outer mesocarp tissues around the calyx and shoulders of the fruit [5]. These “gold specks” are a consequence of the CaOx crystals in the outer mesocarp and can be considered symptoms of Ca excess [6]. Paradoxically, fruits with gold specks can also present BER, which is a typical symptom of calcium deficiency. This apparent contradiction is explained by the distance between the idioblasts with CaOx crystals, arranged at the base of the fruit, and the calcium deficiency in the distal portion, since calcium translocation is a particularly difficult process [4]. The fruit growth rate seems to be an important factor in the incidence of BER, since there is a high demand for calcium in the rapid growth phase of tomato fruits [7]. According to Ho and White [8], BER is initiated by a cellular dysfunction in the distal portion of a young fruit during rapid cell expansion. In this same way, Reitz et al. [9] observed that larger tomato fruits, when compared to smaller fruits of the same age, had an increased incidence of blossom-end rot. This observation reinforces the notion that the mismatch between calcium availability and momentary demand appears to be critical in the development of blossom-end rot.

Due to the arrangement of the fruits on tomato plants, the maximum radiation flux is directed to the basal portion—the shoulder—and this fact allows us to infer that the transpiration rate is higher in this portion than in the distal one. Calcium uptake in tomato plants is highly correlated with solar radiation [10,11]. The higher transpiration rate in the basal portion of fruits may promote a greater xylem flux, increasing the calcium levels, since this ion moves in the xylem sap. There have been reports about the effects of higher transpiration rates increasing calcium in plant tissues [10,12]. Funk and Amatangelo [13], for example, reported that transpiration plays a significant role in determining foliar Ca^2+^ levels in several species of ferns and angiosperms.

Calcium is acquired from the soil solution by the root system and translocated to the shoot through the xylem [2,14]. Several lines of evidence suggest that apoplastic and symplastic pathways contribute to Ca^2+^ delivery to the xylem [2]. When an abundance of calcium is present in the xylem sap, there is a close relationship between transpiration and calcium distribution to the shoot [2]. Thus, as some kinds of fruits are organs with low transpiration rates [2,15], their calcium levels are low. Tomato fruits have a calcium gradient from the basal to distal portions, with lower calcium levels near the apex [10,16]. The precise cause of this unequal calcium distribution is still obscure, but the occurrence of blossom-end rot (BER) in fruits with inadequate xylem development [10,17] suggests that the xylem pattern may be related to calcium distribution. On the other hand, according to Saure [18], the most significant obstacle to the transport of calcium to the fruits seems to be plant mechanisms that restrict its transport, which favors the appropriate fruit growth. During fruit development, the tissue calcium content must remain relatively low during the cell expansion phase to guarantee a high membrane permeability and adequate cell expansion [19].

Calcium oxalate crystals are very common in plant tissues. These crystals have been observed in virtually all plant tissues; they form inside vacuoles of specialized cells called crystal idioblasts [20]. The diversity of CaOx crystal shapes and sizes has led to several hypotheses regarding its function in plants [4,21], including calcium regulation [4,22,23,24], ion balance, plant protection, and detoxification [25].

Since plants are not able to redistribute Ca^2+^ efficiently over long distances, most of the calcium transported through the xylem is utilized or sequestered in the adjacencies of the xylem. Thus, this study aims to evaluate the relationships between calcium levels and the formation of CaOx crystals in wild tomato (*L. pimpinellifolium*) fruits with xylem characteristics in different portions of the pericarp.

## 2. Results

**Figure 1:**
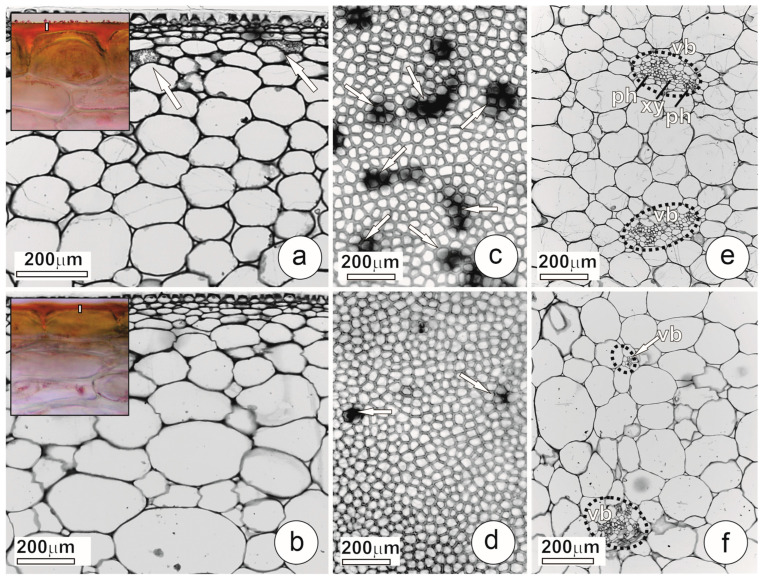
Anatomical structure of *L. pimpinellifolium* mature green fruits. (**a**,**c**,**e**) Details of the fruit basal portion. (**a**) Exocarp in transverse section showing epidermis with thick cuticle and crystal idioblasts (arrows) in epidermal subjacent layers. Note, in detail, the cuticle stained with Sudan red B; the vertical bar inserted into the cuticle is 5 µm high. (**c**) Frontal view of clarified section of outer epidermis; dark spots correspond to crystal idioblasts (arrows). (**e**) Mesocarp in transverse section showing vascular bundles. (**b**,**d**,**f**) Details of the fruit distal portion. (**b**) Exocarp in transverse section showing epidermis with cuticle; note the absence of crystal idioblasts in inner layers. Note, in detail, the cuticle stained with Sudan red B; the vertical bar inserted into the cuticle is 5 µm high. (**d**) Frontal view of clarified section of outer epidermis; note that scarce dark spots correspond to crystal idioblasts (arrows). (**f**) Mesocarp in transverse section showing vascular bundles. (ph: phloem; vb: vascular bundle; xy: xylem).

The epidermis of *L. pimpinellifolium* fruits was uniseriate and showed a thick cuticle (Figure 1a,b), whose thickness decreased towards the distal pole. We did not observe stomata or trichomes (Figure 1c,d).

**Figure 2:**
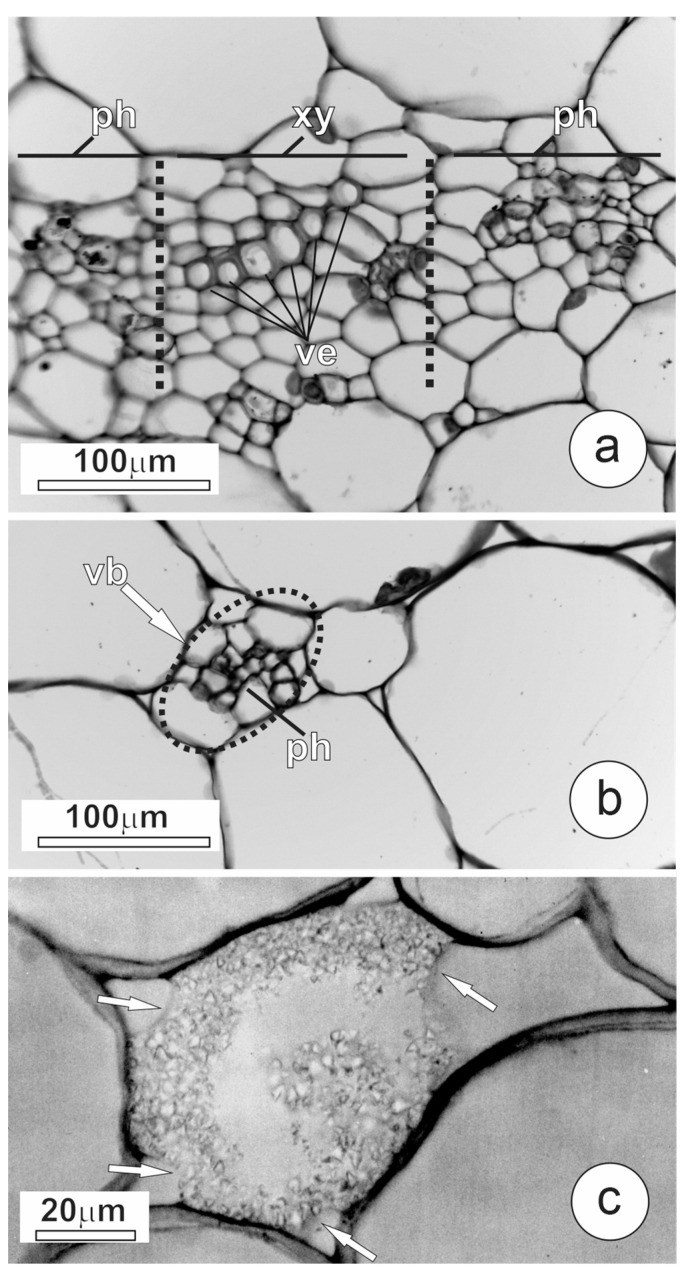
Detail of vascular bundles and crystal idioblast of *L. pimpinellifolium* mature green fruits. (**a**) Transverse section of vascular bundle in the basal portion of the pericarp. Note the presence of large and abundant xylem vessels. The dotted lines simply highlight, approximately, the boundaries between xylem and phloem. (**b**) Transverse section of smaller diameter vascular bundle in the distal pericarp, where xylem is absent. (**c**) Crystal idioblast in the basal pericarp; arrows indicate the thin cell wall. (ph: phloem; vb: vascular bundle; ve: vessel element; xy: xylem).

The pericarp of *L. pimpinellifolium* was predominantly parenchymatous, with large cells showing thin pecto-cellulosic walls, large vacuoles, and some starch grains. The bicollateral vascular bundles were composed of xylem and phloem with their associated parenchymatic cells, while fibers were rare or absent. These bundles showed the xylem in the central portion and two phloem poles disposed towards the outer and inner epidermis (Figure 1e,f and Figure 2a).

**Figure 3:**
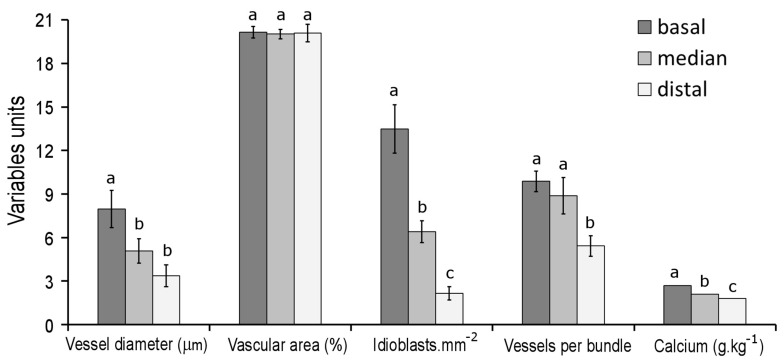
Anatomical data and calcium levels in basal, median, and distal parts of *L. pimpinellifolium* fruit. Different letters above the columns indicate highly significant (*p* < 0.001, one-way ANOVA) differences and which parts of the tomatoes differed from others (Tukey post hoc test). The variable “Vessels per bundle” expresses the average number of tracheary elements in each vascular bundle, for each of the portions of the fruit.

Vascular bundles in the basal portion of the fruit showed large transverse sections (Figure 1e and Figure 2a) and abundant xylem vessels with larger diameters (Figure 2a and Figure 3) than observed in other fruit portions. The composition of vascular bundles in the median portion was similar to that in the basal portion, but the diameter of the vessels was smaller in the median portion (Figure 3).

In the distal pericarp, the vascular bundles were smaller; they showed fewer and narrower xylem vessels (Figure 1f and Figure 2b) compared to other pericarp portions (Figure 3). In the distal portion, xylem vessels were often absent in the bundle ends, which consist exclusively of phloem (Figure 2b). When vascular bundles of the basal and distal pericarp were compared, a reduction in the number and diameter of the vessels was pronounced (Figure 3).

**Figure 4:**
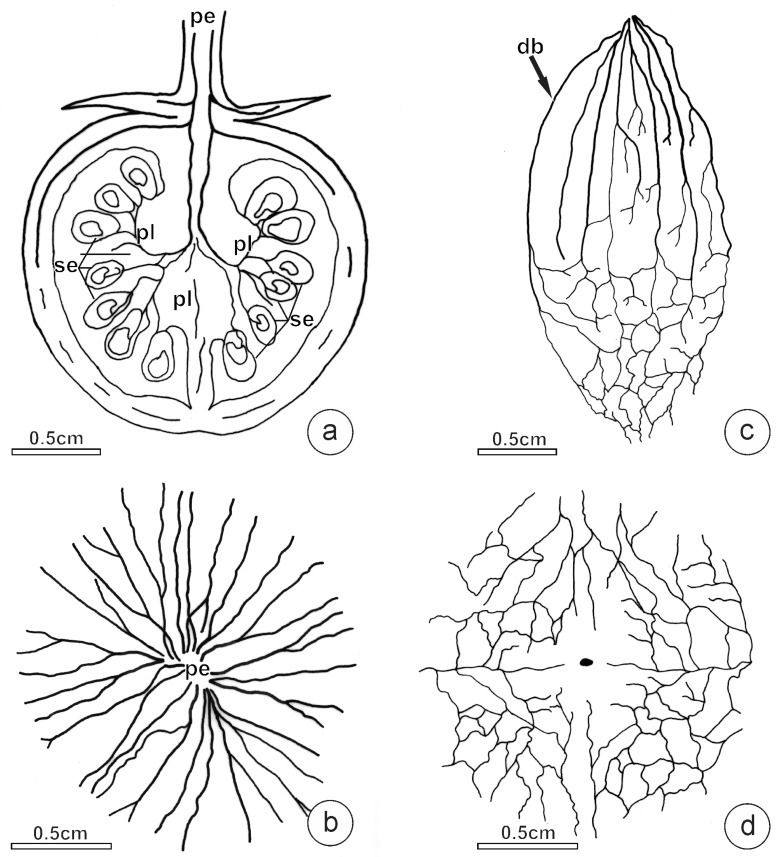
Diagrams showing *L. pimpinellifolium* fruit vascularization. (**a**) Longitudinal section parallel to septum. Note the density of vascular tissues towards the pericarp and placentae portion. (**b**) Frontal view of the clarified basal portion. Note that the vascular bundles are radially disposed and there are few bundle ramifications. (**c**) Clarified pericarp showing vascular bundles. Note the increase in bundle ramifications from the basal (**upper**) to the distal portion (**lower**). (**d**) Frontal view of the clarified distal portion. Note the intense ramification of vascular bundles. (db: dorsal bundle; pe: peduncle; pl: placentae; se: seed).

At the transition between the peduncle and pericarp, the vascular bundles divided into two branches: the outer vascular bundles vascularized the pericarp, and the inner ones were directed to the placentae (Figure 4a). In the basal pericarp, the vascular bundles irradiated from the peduncle insertion point, presented few ramifications, and were predominantly straight (Figure 4b). In the median pericarp, ramifications of vascular bundles were observed, which became numerous towards the distal pericarp, where these highly ramified bundles join, forming an anastomosed system (Figure 4c,d). The density of the vascular tissue, estimated by the proportion of the occupied area in the frontal view, was similar in these three studied fruit portions (Figure 3; compare Figure 4b and Figure 4d).

The calcium concentration decreased from the fruit basal portion to the distal one (Figure 3). The CaOx crystals occurred in crystal idioblasts in the outer mesocarp, especially in the subepidermal layers (Figure 1e,f and Figure 2c). These cells showed thin pectin-rich walls and vacuoles full of CaOx as sand crystals. Since many of these idioblasts have disrupted the wall, the crystals seem free in the intercellular spaces, creating a false impression about crystal localization. The idioblasts were more abundant in the basal portion of the fruit, decreasing towards the distal end (Figure 1c,d and Figure 3).

## 3. Discussion

As reported for tomato fruits (*Lycopersicon esculentum*) [26], there were no stomata in the fruits of *L. pimpinellifolium*, which were also characterized by an epidermis with a thicker cuticle. Both traits constitute barriers to transpiration and support the low transpiration rate commonly attributed to this organ. Applying ^45^Ca to orange (*Citrus sinensis*) fruits, Bonomelli et al. [27] observed higher rates of calcium transport into the pulp during the fruit set in contrast to the other stages of fruit development. It is known that young organs are subject to a higher transpiration rate because they do not have structural defenses against excessive water loss, especially a cuticle that is not yet fully formed. The cuticle composition changes during tomato fruit development, increasing the density and thickness 15 days after anthesis [28]. Although our study has been restricted to mature green fruits, which are fully developed from a structural perspective, considering the dynamics of calcium supply in the earlier stages of development is essential for understanding the role of xylem in calcium distribution. It is worth noting that the low translocation of calcium in the distal portions of the shoot results in the accumulation of this nutrient over the phases preceding the analysis.

The vascular bundles in *L. pimpinellifolium* pericarp are bicollateral, which is characteristic of the Solanaceae. Despite the marked changes that we observed occurring in the composition of the vascular bundles along the fruit length, the density of the vascular system (vascular area in the frontal view) showed no differences. This stability in the density of the vascular system may be a consequence of the high bundle ramifications that occurred in the distal fruit portions, which compensate for the reduction in the bundle area. It is important to emphasize that although we observed vascular tissues well distributed throughout the fruit length, the bundle composition presented relevant differences. In the fruit distal portion, the number of xylem vessels per vascular bundle and the average vessel diameter were half those in the basal portion. These key traits of the xylem into the vascular bundles were coherent with the gradient of calcium levels and the density of CaOx crystal idioblasts.

The xylem reduction we observed along the fruit length resulted in a lower proportion of this tissue in the distal portions, where phloem became prevalent. These vascular bundle changes seem to be coherent with the higher incidence of BER in the varieties with longer fruits than those with shorter fruits [29]. Our results suggest that differences in the xylem/phloem ratio of the vascular bundle in long fruits tend to be greater and impact calcium distribution throughout the fruit.

In *Lycopersicon esculentum*, the number of vascular bundles in the basal fruit portion was greater than in the distal portion [30]; however, the authors have not described the bundle details, such as the vessel diameter and number, even though these parameters are important for estimating the xylem sap flow. According to Ho et al. [30], the density of the vascular system per cross-sectional area of fruit can be used to measure the relative capacity of xylem transport. The poor transport of calcium in the fruit may be the cause of the lower Ca concentration in the extracellular calcium pool of the distal fruit portion than in other portions.

Low calcium levels in a plant organ are not always the result of insufficient calcium absorption but may be a consequence of an unbalanced calcium distribution. Our data show a relationship between calcium levels and xylem structure in fruit, reflecting the occurrence of CaOx crystal idioblasts. According to Paiva et al. [12], high transpiration rates in fruit stimulate xylem transport and increase calcium levels, as previously demonstrated by Wiersum [31].

Calcium oxalate crystals in the pericarp of *L. pimpinellifolium* may act as a drain for calcium excess, regulating calcium cytosolic levels, as suggested by several authors (see [32] and reference therein). Macnish et al. [33] stated that in *Chamelaucium uncinatum* (Myrtaceae), CaOx crystals may regulate soluble calcium concentrations near sites where this ion is unloaded from the xylem. Calcium entry into many plants is not necessarily related to their metabolic requirements but rather to substrate concentrations and the calcium permeability of the apoplastic pathway to the xylem in roots [2]; this fact may justify the calcium excess.

The lowest calcium content in the distal portion of *L. pimpinellifolium* fruit follows Adams and Ho’s [10] and Nonami et al. [34] observations in tomato (*Lycopersicon esculentum*). Our data also showed a relationship between calcium and the number of xylem vessels, since both showed an evident pattern of decreasing from the basal portion to the distal portion of the fruits. According to Simon [35], fruits only receive an initial supply of calcium via the xylem, and after the first stage of development, transpiration rates fall; then, a later input of nutrients enters via the phloem, in which calcium is relatively immobile. Thus, a decrease in the calcium levels from the proximal to the distal end of the *L. pimpinellifolium* could be expected, once it was reported by Saure [18] as a common trait in fruits.

Developing organs can receive significant amounts of water from the phloem [31,36]. Phloem is important in water transport to the tomato fruits [37] but seems to be not efficient for calcium transport to meet fruits requirements. Inadequate xylem development was pointed out by Taylor and Locascio [17] as one cause of blossom end-rot due to poor calcium nutrition. Therefore, it is necessary to consider that the initial supply of calcium in fruits is important to establish an adequate reserve of this nutrient, making it available for phases of rapid expansion, during which the supply via the xylem does not always occur at satisfactory rates [31,38].

Our data show a decreasing gradient of crystal idioblasts from the basal portion towards distal fruit portions in *L. pimpinellifolium* pericarp. Considering that the calcium levels in the fruit have peculiar distribution patterns, we consider the existence of a positive relationship between calcium levels and crystal formation, as previously described by Zindler-Frank et al. [39] and Volk et al. [22] in other species and plant organs.

Although the density of the vascular bundle did not differ between the basal and distal fruit portions in *L. pimpinellifolium*, the calcium level differences observed in these portions may be due to the vascular bundle composition. In the distal portion, vascular bundles have smaller diameters, and the xylem consists of a few narrow vessel members that can be absent in some vascular ends. The occurrence of CaOx crystals in cells adjacent to the vascular bundles is common [24], and this spatial crystal pattern suggests a role for CaOx in regulating calcium transport [40]. However, in the wild tomato fruits analyzed, the crystal idioblasts accumulate in the superficial layers of the mesocarp, near the epidermis, and are not associated with vascular bundles in a pattern similar to that observed in tomato *Lycopersicon esculentum* fruits [5]. This distribution pattern appears to reflect the low concentration of calcium in the fruits and, above all, the prevalence of transpiration directing apoplastic transport towards the surface of the fruit shoulder. The formation of CaOx crystals in the vicinity of vascular tissues is common in organs with a high supply of calcium, such as in leaves [24].

In addition, the differences between calcium levels in *L. pimpinellifolium* fruits may be attributed to a problem with calcium transport, as proposed by Ehret and Ho [41]. Our results show that the differences in vascular bundle anatomy, especially in the xylem composition, may be correlated with calcium distribution. In this same way, Winkler et al. [42] concluded that “xylem functionality is the primary determinant of the Ca relations of sweet cherry fruit”. It is important to emphasize that although xylem contribution to the water and nutrient influx decreases during tomato fruit development [31,37], this reduction in xylem flow seems to result from increasing the fruit’s defenses against excessive transpiration. In the anatomical analyses, we did not observe any anomaly in the tracheal elements, so that any reduction in transport must result from factors that influence transpiration. We need to take into account that even if xylem functionality is preserved, the decrease in the number of tracheal elements, and particularly their absence in the vascular endings, undoubtedly contributes to a deficiency in calcium distribution. The anatomical analyses reinforce the observation of Hanssens et al. [11], who reported the continued functionality of the xylem pathway in tomato fruits even at 35 days after anthesis. In our study, we detected no morphological alterations indicative of water flow obstruction within the tracheal elements until the fruit ripening stage. It follows that the decrease in transpiration and the distribution of vascular elements emerge as the predominant structural factors explaining the calcium distribution in wild tomato fruits.

## 4. Materials and Methods

Mature green fruits of wild tomato (*Lycopersicon pimpinellifolium* L.) Mill. plants growing individually in pots containing the same soil type were sampled for the structural and calcium level studies. For light microscopy, pericarp samples from basal, median, and distal fruit portions were taken; they were fixed in Karnovsky mixture [43], processed, and embedded in 2-hydroxyethyl-methacrylate (Leica Embedding Kit); 5 µm thick transverse and longitudinal sections were obtained with a rotary microtome. The sections were distended on slides and stained with toluidine blue O [44]. Hand-cut sections were obtained with a razor blade and submitted to the following histochemical tests: ruthenium red to detect acid polysaccharides [45], Sudan Red 7B for total lipids (modified from [46]), and Lugol for starch [45]. All sections were observed under a CX41RF microscope (Olympus Scientific Solutions, Waltham, MA, USA) equipped with a digital camera and image capturing system (TV0.5XC-3, Olympus Scientific Solutions, Waltham, MA, USA).

The density of the vascular system was estimated via direct measurement of the vascular bundles area in clearing pericarp, analyzed in frontal view. Pericarp samples were cleared following Fuchs [47], and the anatomical data were obtained via measurements under light microscopy. The density of the vascular system was expressed as the ratio between the area occupied by the vascular bundles and the total area. Five fruits from different individuals and ten microscope fields within each fruit portion were evaluated for statistical analysis. All measured variables showed normality (Shapiro–Wilk test) and homoscedasticity (Bartlett test), being submitted to one-way ANOVA followed by a post hoc Tukey test when significant differences were detected. For illustration purposes, the vascular system of each studied portion was drawn using a drawing tube adapted to a light microscope (BH-2; Olympus Optical, Tokyo, Japan).

For calcium analysis, pericarp portions from 25 fruits were sampled at basal, median, and distal portions. The analyses were carried out on three samples composed of a mixture from these 25 fruits. Septa, placentae, and seeds were not analyzed. The ground-milled dry material underwent wet acid digestion with nitric and perchloric acids, and calcium contents were determined using flame atomic absorption spectrophotometry (Varian SpectrAA 220 FS, Varian, Palo Alto, CA, USA).

## 5. Conclusions

Using wild tomato (*Lycopersicon pimpinellifolium*) fruits as a model, we demonstrated that there is a clear relationship between the composition of vascular tissues and the presence of calcium along with CaOx crystals. Such interdependence is likely associated with physiological disorders related to imbalances in calcium levels, whether due to an excess or deficiency of this nutrient. The basal portion of the wild tomato fruit exhibits vascular bundles with abundant and large xylem vessels. Conversely, in the distal pericarp, the vascular bundles are smaller and show fewer and narrower xylem vessels, often absent in the bundle ends. Our findings further indicate that variations in xylem structure and the xylem/phloem ratio within the vascular bundle impact the calcium distribution throughout the fruit. Considering the interaction between the vascular system and calcium distribution, efforts should be made to better understand the interactions between calcium-related disorders and the structure of vascular tissues.

## Data Availability

The data presented in this study are available on request from the corresponding author.
